# Transplacental Passage of Interleukins 4 and 13?

**DOI:** 10.1371/journal.pone.0004660

**Published:** 2009-03-02

**Authors:** Robert H. Lim, Lester Kobzik

**Affiliations:** 1 Department of Environmental Health, Harvard School of Public Health, Boston, Massachusetts, United States of America; 2 Division of Respiratory Diseases, Children's Hospital Boston, Boston, Massachusetts, United States of America; 3 Department of Pathology, Brigham & Women's Hospital, Boston, Massachusetts, United States of America; University of Toronto, Canada

## Abstract

The mechanisms by which prenatal events affect development of adult disease are incompletely characterized. Based on findings in a murine model of maternal transmission of asthma risk, we sought to test the role of the pro-asthmatic cytokines interleukin IL-4 and -13. To assess transplacental passage of functional cytokines, we assayed phosphorylation of STAT-6, a marker of IL-4 and -13 signaling via heterodimeric receptor complexes which require an IL-4 receptor alpha subunit. IL-4 receptor alpha−/− females were mated to wild-type males, and pregnant females were injected with supraphysiologic doses of IL-4 or 13. One hour after injection, the receptor heterozygotic embryos were harvested and tissue nuclear proteins extracts assayed for phosphorylation of STAT-6 by Western blot. While direct injection of embryos produced a robust positive control, no phosphorylation was seen after maternal injection with either IL-4 or -13, indicating that neither crossed the placenta in detectable amounts. The data demonstrate a useful approach to assay for transplacental passage of functional maternal molecules, and indicate that molecules other than IL-4 and IL-13 may mediate transplacental effects in maternal transmission of asthma risk.

## Introduction

Prenatal events can affect development of certain adult diseases [1]–[4], but the mechanisms are not clear. For example, maternal atopy/allergy can predispose offspring to the development of asthma [5], [6], more so than paternal disease. Moreover, there is evidence in mice and humans that allergic sensitization may occur in the prenatal period[7]–[9]. Our laboratory has developed a mouse model that recapitulates this ‘maternal effect’[10]. In this model, offspring of mother mice with ovalbumin (OVA)-induced asthma develop an asthma-like phenotype (i.e. airway hyperreactivity and airway inflammation) following an ‘intentionally suboptimal’ asthma induction protocol, whereas offspring of non-asthmatic mother mice do not. Importantly, this maternal effect is allergen-independent, since offspring show increased susceptibility to other allergens besides OVA. This suggests a role for mediators with broad effects, e.g., cytokines, rather then specific antibodies.

These data taken together with prenatal sensitization observations, imply that the *in utero* environment may be causing increased asthma risk in offspring. One possible mechanism by which maternal asthma could cause increased asthma susceptibility in the developing immune system is the transplacental passage of ‘pro-allergic’ cytokines.

In addition to providing the fetus with oxygen and nutrients, the placenta synthesizes and secretes hormones, growth factors, and cytokines. Moreover, many maternally-derived molecules cross the placental via a variety of mechanisms [11]. Based on findings in our mouse model of maternal transmission, we postulated that passage of pro-asthmatic maternal cytokines could mediate increased susceptibility of offspring to asthma.

The aim of this study was to characterize a novel assay to assess for the transplacental passage of functional cytokines, and to use this technique to assess for passage of the pro-asthmatic/pro-inflammatory cytokines interleukin 4 and 13 (IL-4 and IL-13) previously implicated in maternal transmission of asthma risk [10], [12].

## Results

In order to devise a functional assay to assess for transplacental passage of IL-4 and 13, we made use of IL4 receptor alpha (IL4Ra)−/− transgenic mice and STAT-6 phosphorylation. IL-4 acts rapidly and primarily through phosphorylation of STAT-6 via binding to IL-4 receptor type I or II. The type I receptor is a heterodimer made up of an IL4Ra and gamma-chain subunits. The type II receptor is made up of IL4Ra and IL-13 receptor alpha 1 (IL13Ra1) subunits. IL-13 acts through the type I IL-4 receptor. However IL-13 binds to the IL13Ra1 subunit rather then the IL4Ra subunit (reviewed [13]). Following binding, STAT6 is phosphorylated, dimerizes, and translocates to the nucleus. Disruption of the IL-4Ra gene can prevent the phosphorylation of STAT-6 caused by either IL-4 or 13.

In order to assess if functional IL-4 or 13 can cross the placenta, this study used female IL4Ra−/− transgenic mice. These mice were mated to wild-type males. Thus the resulting embryos were IL4Ra+/−. On day 20 of pregnancy, the female mice were given retro-orbital injections of IL-4 or 13. As the mothers were IL-4Ra−/−, there would be no signaling by the cytokines (i.e., phosphorylation of STAT-6) and no downstream effects that might confuse interpretation (i.e. signaling-induced release of other mediator(s) which cross the placenta and cause effects rather than the IL-4 or IL-13 per se). However, since the embryos are IL4Ra+/−, they can respond to IL-4 and 13. Thus, if phosphorylation of STAT-6 is detected in fetal tissue, then the cytokines would have had to have crossed the placenta in a functionally intact state to interact with the IL-4Ra present on the heterozygotic fetal cells. The phosphorylation signal is detectable by Western blot analysis using a phospho-STAT-6 (P-STAT-6) antibody.

### Injection of IL-4 and IL-13 causes detectable phosphorylation of STAT-6 in fetal lung nuclear protein extract, but not total protein extracts

Following retro-orbital (R.O.) or intraperitoneal injection (I.P.) of IL-4 or IL-13 into wild type adults, Western blot analysis showed ample phosphorylation of STAT-6 in lung tissue total protein extracts. Similar results were observed using IL4Ra+/− adults. Non-injected mice showed no phosphorylation. After having demonstrated that retro-orbital and intraperitoneal injections had similar results, further adult mouse injections were all retro-orbital. We were unable to clearly and consistently detect phosphorylation of STAT-6 in lung tissue total protein extracts of directly injected (intraperitoneal injections) embryos (either wildtype or heterozygote) (not shown). The embryos were given I.P. injections because retro-orbital injections were not technically feasible.

**Figure 1:**
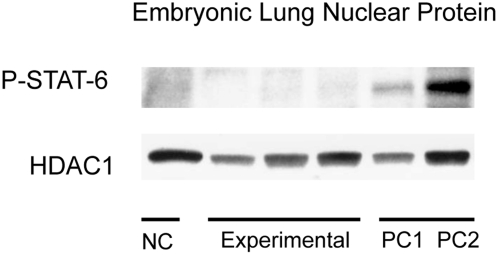
STAT-6 phosphorylation following injection with IL-4. Positive control consists of lung nuclear proteins from wildtype adults (PC1) or IL4Ra+/−embryos directly injected with IL-4 (PC2). Negative controls consist of lung nuclear protein from IL4Ra+/− embryos directly injected with PBS (NC). The experimental group consists of nuclear proteins from IL4Ra+/− embryos whose mothers were injected with IL-4. Bands for P-STAT-6 are only detectable in positive control lanes. No bands are detected in experimental and negative control lanes. HDAC1 was used a loading control.

**Figure 2:**
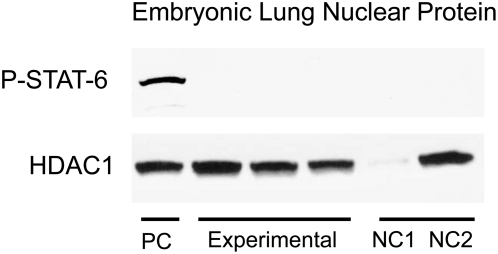
STAT-6 phosphorylation following injection with IL-13. Positive control (PC) consists of lung nuclear proteins from IL4Ra+/− embryos directly injected with IL-13. Negative controls consist of lung nuclear protein from IL4Ra−/− females injected with IL-13 (NC1) and nuclear protein from IL4Ra+/− embryos injected with PBS (NC2). The experimental group consists of nuclear proteins from IL4Ra+/− embryos whose mothers were injected with IL-13. Bands for P-STAT-6 are only detectable in PC lane. HDAC1 was used as a loading control. Insufficient protein was loaded in NC1, but otherwise protein loading was similar.

In order to increase our ability to detect phosphorylation of STAT-6, nuclear protein extracts from lung tissue were used. As STAT-6 is translocated into the nucleus following phosphorylation, the nuclear fraction of protein is enriched with the phosphorylated form. Using nuclear protein extracts from lung tissue, phosphorylation of STAT-6 could be detected following injection of IL-4 into adult wild-type mice (R.O.) and fetal (I.P.) IL4Ra+/− mice (Figure 1, PC1 and PC2, respectively). An appropriate size band for phosphorylated STAT-6 is seen in both positive control lanes. No phosphorylation was seen in PBS injected specimens (Figure 1, NC). Similar data was seen using IL-13 injections (Figure 2, PC and NC1, NC2). HDAC1 was used a loading control.

### Absence of evidence that IL-4 or IL-13 cross the placenta

Results for the IL-4 maternal injections are summarized in figure 1. The negative controls were nuclear protein extracts from pooled lungs from 3 IL4Ra+/− embryos injected (I.P.) with PBS. The positive controls were nuclear extracts from lungs of a wild-type female and pooled lungs from 3 IL4Ra+/− fetuses injected with IL-4, respectively. The experimental group consisted of nuclear protein extracts from pooled lung tissue from 3 IL4Ra+/− fetuses after maternal injection (R.O.) of IL-4. Maternal injections were done in a total of 9 pregnant females. Figure 1 shows representative data from 3 trials. In negative control lanes, no bands were detected for phosphorylated STAT-6 (Figure 1). Appropriate size bands were noted in both positive control lanes (Figure 1). In the 3 experimental lanes, there were no detectable phosphorylated STAT-6 bands. From this data, it can be concluded that sufficient functional IL-4 did not cross the placenta. HDAC1 was used as a nuclear protein loading control.

In a second of set of experiments, IL-13 was injected into IL4Ra−/− pregnant females (R.O.). The results were similar to those found using IL-4. Maternal injections were done in a total of 9 pregnant female. Figure 2 shows representative data from 3 of the 9 injections. Heterozygote fetuses of injected mothers did not show phosphorylation of STAT-6, though it was seen in directly injected (I.P.) heterozygote fetuses (Figure 2). Both negative controls in figure 2 do not show a band, though NC1 had significantly less nuclear protein loaded as compared to others. From this blot, it could be concluded that sufficient functional IL-13 did not cross the placenta.

## Discussion

In order to assess for *in vivo* transplacental passage of functional cytokines, we devised a novel technique using IL4Ra−/− mice. These female mice were mated with wild-type males, producing heterozygote embryos. The mothers were injected with supraphysiologic doses of IL-4 or IL-13, and the nuclear extracts of the embryonic lungs were analyzed using Western Blot for presence of phosphorylated STAT-6. Both IL4 and IL13 act via binding to a receptor heterodimer complex that includes an IL4Ra subunit. Following binding, Janus family of protein kinases (Jaks) that are constitutively associated with IL-4Ra are activated. The end result of Jak and tyrosine kinase activation is STAT-6 phosphorylation [13]. Because the mothers lacked IL4Ra, and are unable to phosporylate STAT-6 in response to either IL-4 or 13, then any phosphorylation of STAT-6 in the heterozygote embryos would be a result of transplacental passage of functional cytokines. Aside from these canonical activators of STAT-6, there may also be some alternative pathways [14]. Some studies have shown that IL-3 [15], 15 [16], platelet derived growth factor [17], and IFN-gamma [18] can activate STAT-6, though these experiments were done in cell lines and may not be relevant to this experimental model, especially given the negative results.

In order to maximize the likelihood of placental crossing, the pregnant IL4Ra−/−females in the experimental groups were injected with supraphysiologic doses of IL-4 and IL-13 (1000 ng). Based on the lack of phosphorylated STAT-6 found in heterozygote embryos, it is unlikely that either IL-4 or IL-13 cross the placenta at the dose used. We also interpret the data to indicate that it is unlikely that either cytokine would cross under natural conditions as the injected dose was much higher the normal serum ranges.

It is possible that gestational age at time of cytokine delivery may affect its transplacental passage, and that at earlier time-points (i.e. <20 days gestation) cytokine may be able to cross the placenta. However studies examining the relationship of gestational age and placental permeability to cytokines are lacking. From a practical standpoint, this experimental approach is only feasible at gestational ages greater than 18 days. At earlier gestational ages, harvesting of lung tissue becomes problematic. Due to the size and consistency of the embryo, it is difficult to reliably isolate sufficient amounts of lung tissue and separate the heart-lung block. Direct embryonic intraperitoneal injection also becomes increasingly challenging with earlier gestational ages making generation of appropriate positive and negative controls difficult. Hence, this experimental approach may be most useful in assaying transplacental passage of maternal molecules in the near term embryo.

Lung tissue is not the only tissue that could have been targeted for analysis. The lung was chosen as STAT-6 is known to play a key role in pulmonary eosinophilia and airway hyperreactivity in the context of experimental asthma [19]–[21]. Others have also examined STAT-6 activation in lung tissue[22]. Another easily accessible target embryonic organ is the liver. In pilot experiments, we attempted to use liver tissue, however were unable to obtain clear consistent signal in positive controls, as opposed to in lung tissue.

Several potential problems to this study merit discussion. The first is the somewhat arbitrary nature of the 1 hour time point picked for time from injection of mother until embryo harvest. This time point was used since in an *ex vivo* perfusate study examing transplacental passage of IL-6, evidence for passage was found in less then 1 hour [23]. Our data do not exclude the possibility that both or one of these cytokines cross the placenta, in a process that takes longer then 1 hour. A second potential issue is the relatively short half-life of phosphorylated STAT-6. In cell culture studies, the half-life of phosphorylated STAT-6 after a single bolus dose of IL-4 was less then one hour. However, phosphorylated STAT-6 persisted much longer with a more prolonged exposure to IL-4 [24]. It is possible that even if transplacental passage of IL-4 or 13 occurred, phosphorylated STAT-6 may have already degraded by the time the embryos were harvested, leading to a false negative. This seems unlikely as the positive controls (both embryo and adult) still showed phosphorylation of STAT-6 at the same 1 hour time point. A 3^rd^ possible problem is the sensitivity of Western Blot for detection of phosphorylated -STAT-6. If only a small amount of IL-4 or 13 cross the placenta, then the level phosphorylation may be very low, and not detectable by Western Blot. While this is a possibility, given that our positive controls (directly injected ILRa+/− embryos) showed phosphorylation, this does not seem likely.

The maternal-fetal interface is an immunologically active site rich in cytokines [25]–[27]. However, few studies have looked specifically at the transplacental passage of cytokines. The published studies use two approaches. The first relies on injection of radiolabelled cytokines into mothers and assessing the offspring for radioactivity [28]. The draw back of this approach is that even if radioactivity is detected in the offspring, it is possible that just a metabolized fragment of the cytokine containing the radioactive tag crossed, rather than a functional intact cytokine. The second type of study is performed by perfusing placentas *ex vivo*. The drawbacks of these studies are the technical difficulties and the arguably non- physiologic system. This approach is also not practical for mouse studies. Obviously, neither system is ideal. Moreover, results with these approaches have been quite variable[23], [29]–[32], with data both supporting (e.g. [23], [31]) and refuting (e.g.[29], [30], [32]) transplacental passage. The approach used in this study to assess for transplacental passage of functional cytokine is a unique extension of r the one other study we found in the literature that used a similar approach. Use of CSF-1 deficient mice (csfm^op^/csfm^op^) demonstrated that heterozygote mothers (+/csfm^op^) could pass sufficient CSF-1 across the placenta to promote the normal appearance of macrophages in csfm^op^/csfm^op^ embryos[33]. However, that study did not test for functional effects directly related to maternally injected cytokines.

Overall, our study illustrates the potential of this functional approach to evaluate transplacental passage of functional mediators. This approach does require that mediators in question operate through a receptor for which viable, reproductively normal, receptor-deficient mice exist. This is true for many, but not all, potential molecules of interest. For our maternal transmission model, we conclude that the data do not support the postulate that IL-4 and 13 cross the placenta under these experimental conditions. This result was surprising in that our previous work implied a key role for maternal IL-4 in the maternal transmission of asthma risk [10]. That study demonstrated that maternally injected monoclonal IL-4 antibody could attenuate offspring asthma susceptibility. The negative finding from this study has stimulated ongoing studies of other potential mediators, e.g. stress hormones. Although the results of this study were negative, this experimental approach using transgenic mice may be useful in future studies that assess for transplacental passage of maternal molecules.

## Materials and Methods

Male and female BALB/c mice, 8–10 week-old were obtained commercially from Charles River Laboratories (Wilmington, MA). IL-4 receptor alpha knockout (IL4RaKO) mice (BALB/c background) were obtained from Jackson Laboratories (BALB/c-Il4ra^tm1Sz^/J, stock number 003514). Mice were housed and fed standard lab chow *ad libitum* in a pathogen-free barrier facility that was maintained at 22–24°C with a 12-h dark/light cycle. Animal experiments and husbandry standards were approved by the Harvard Medical Area Standing Committee on Animals.

8–10 week old female IL4Ra−/− mice were mated with wild-type BALB/c mice. At approximately 20 (E20) days post-mating, the pregnant females were anesthetized with an intramuscular injection of ketamine/xylazine. Once adequate anesthesia was obtained, the females were given a retro-orbital injection of IL-4 or IL-13 (1000 ng, Peprotech). One hour after injection, the mothers were euthanized. The heterozygote embryo lungs were harvested as were the maternal lungs. The lungs were flashed frozen in liquid nitrogen and stored at −80 degrees C.

For positive and negative controls, a subset of the pregnant females were processed differently. The E20 females were anesthetized with ketamine/xylazine. Then a 1 cm incision was made in the abdomen. The embryos were then exposed. In a litter, half the fetuses were directly injected (I.P.) with IL-4 or IL-13 (500 ng, positive controls) or PBS (negative controls). The embryos were then re-inserted into the mothers abdomen. The fetal lungs were then harvest an hour later. In the interim between injection and harvest, the mother's abdominal cavity was periodically washed with warm (37 degree Celsius) PBS to maintain maternal temperature and to prevent dessication of the abdominal cavity and fetuses. At time of harvest, fetuses were confirmed to be alive by fetal movement prior to euthanasia.

Nuclear protein was extracted from frozen lungs. The tissue was homogenized in lysis buffer (1.5 mM MgCl2, 10 mM KCl, 0.1% NP-40, 0.5 nM DTT, Roche complete protease inhibitor (1×), and 10 mM HEPES (pH 7.9)). The lysate was then centrifuged at 900 RCF for 10 minutes at 4 degrees C. Supernatant was removed and pellet resuspended in the wash buffer. This was centrifuged again at 1200 RCF for 10 minutes at 4 degrees C. Supernatant was discard and resuspended in nuclear extraction buffer (1.5 mM MgCl2, 0.42 M NaCl, 0.2 mM EDTA (pH 8.0), 25% glycerol, 0.5 M DTT, and 20 mM HEPES (pH 7.9)). This was mixed on an orbital shaker for 30–60 minutes, then spun down at 20,000 RCF for 15 minutes at 4 degrees C. The supernatant, which constituted the nuclear protein, was then aliquoted and frozen at −80 degrees. This amount of protein was quantified using a bicinchoninic acid assay (BioRad). Western Blots were performed using a Tris-Glycine Gel System (Invitrogen). Membranes were probed using polyclonal antibodies raised against Phospho-STAT-6 (PSTAT6) and HDAC1 (Santa Cruz Biotechnologies, Cell Signaling Technologies). Primary antibodies were detected with HRP conjugated anti-rabbit polyclonal antibody. Bands were visualized using the ECL+ Western Blotting Detection System (Amersham) and the Alpha-Innotech gel imager.

## References

[1] Franzek EJ, Sprangers N, Janssens AC, Van Duijn CM, Van De Wetering BJ (2008). Prenatal exposure to the 1944–45 Dutch ‘hunger winter’ and addiction later in life.. Addiction.

[2] Painter RC, Osmond C, Gluckman P, Hanson M, Phillips DI (2008). Transgenerational effects of prenatal exposure to the Dutch famine on neonatal adiposity and health in later life.. BJOG.

[3] de Rooij SR, Painter RC, Phillips DI, Osmond C, Tanck MW (2006). Cortisol responses to psychological stress in adults after prenatal exposure to the Dutch famine.. Psychoneuroendocrinology.

[4] St Clair D, Xu M, Wang P, Yu Y, Fang Y (2005). Rates of adult schizophrenia following prenatal exposure to the Chinese famine of 1959–1961.. JAMA.

[5] Rees J (2005). ABC of asthma. Prevalence.. Bmj.

[6] Litonjua AA, Carey VJ, Burge HA, Weiss ST, Gold DR (1998). Parental history and the risk for childhood asthma. Does mother confer more risk than father?. Am J Respir Crit Care Med.

[7] Devereux G, Seaton A, Barker RN (2001). In utero priming of allergen-specific helper T cells.. Clin Exp Allergy.

[8] Herz U, Ahrens B, Scheffold A, Joachim R, Radbruch A (2000). Impact of in utero Th2 immunity on T cell deviation and subsequent immediate-type hypersensitivity in the neonate.. Eur J Immunol.

[9] Herz U, Joachim R, Ahrens B, Scheffold A, Radbruch A (2000). Prenatal sensitization in a mouse model.. Am J Respir Crit Care Med.

[10] Hamada K, Suzaki Y, Goldman A, Ning YY, Goldsmith C (2003). Allergen-independent maternal transmission of asthma susceptibility.. J Immunol.

[11] Fuchs R, Ellinger I (2004). Endocytic and transcytotic processes in villous syncytiotrophoblast: role in nutrient transport to the human fetus.. Traffic.

[12] Hubeau C, Apostolou I, Kobzik L (2006). Adoptively transferred allergen-specific T cells cause maternal transmission of asthma risk.. Am J Pathol.

[13] Chatila TA (2004). Interleukin-4 receptor signaling pathways in asthma pathogenesis.. Trends Mol Med.

[14] Wurster AL, Tanaka T, Grusby MJ (2000). The biology of Stat4 and Stat6.. Oncogene.

[15] Quelle FW, Shimoda K, Thierfelder W, Fischer C, Kim A (1995). Cloning of murine Stat6 and human Stat6, Stat proteins that are tyrosine phosphorylated in responses to IL-4 and IL-3 but are not required for mitogenesis.. Mol Cell Biol.

[16] Qin JZ, Zhang CL, Kamarashev J, Dummer R, Burg G (2001). Interleukin-7 and interleukin-15 regulate the expression of the bcl-2 and c-myb genes in cutaneous T-cell lymphoma cells.. Blood.

[17] Patel BK, Wang LM, Lee CC, Taylor WG, Pierce JH (1996). Stat6 and Jak1 are common elements in platelet-derived growth factor and interleukin-4 signal transduction pathways in NIH 3T3 fibroblasts.. J Biol Chem.

[18] Fasler-Kan E, Pansky A, Wiederkehr M, Battegay M, Heim MH (1998). Interferon-alpha activates signal transducers and activators of transcription 5 and 6 in Daudi cells.. Eur J Biochem.

[19] Akimoto T, Numata F, Tamura M, Takata Y, Higashida N (1998). Abrogation of bronchial eosinophilic inflammation and airway hyperreactivity in signal transducers and activators of transcription (STAT)6-deficient mice.. J Exp Med.

[20] Hoshino A, Tsuji T, Matsuzaki J, Jinushi T, Ashino S (2004). STAT6-mediated signaling in Th2-dependent allergic asthma: critical role for the development of eosinophilia, airway hyper-responsiveness and mucus hypersecretion, distinct from its role in Th2 differentiation.. Int Immunol.

[21] Kuperman D, Schofield B, Wills-Karp M, Grusby MJ (1998). Signal transducer and activator of transcription factor 6 (Stat6)-deficient mice are protected from antigen-induced airway hyperresponsiveness and mucus production.. J Exp Med.

[22] Hirota JA, Ask K, Fritz D, Ellis R, Wattie J (2008). Role of STAT6 and SMAD2 in a model of chronic allergen exposure: a mouse strain comparison study.. Clin Exp Allergy.

[23] Zaretsky MV, Alexander JM, Byrd W, Bawdon RE (2004). Transfer of inflammatory cytokines across the placenta.. Obstet Gynecol.

[24] Mattes J, Yang M, Siqueira A, Clark K, MacKenzie J (2001). IL-13 induces airways hyperreactivity independently of the IL-4R alpha chain in the allergic lung.. J Immunol.

[25] Bowen JM, Chamley L, Mitchell MD, Keelan JA (2002). Cytokines of the placenta and extra-placental membranes: biosynthesis, secretion and roles in establishment of pregnancy in women.. Placenta.

[26] Ostojic S, Dubanchet S, Chaouat G, Abdelkarim M, Truyens C (2003). Demonstration of the presence of IL-16, IL-17 and IL-18 at the murine fetomaternal interface during murine pregnancy.. Am J Reprod Immunol.

[27] Zourbas S, Dubanchet S, Martal J, Chaouat G (2001). Localization of pro-inflammatory (IL-12, IL-15) and anti-inflammatory (IL-11, IL-13) cytokines at the foetomaternal interface during murine pregnancy.. Clin Exp Immunol.

[28] Letterio JJ, Geiser AG, Kulkarni AB, Roche NS, Sporn MB (1994). Maternal rescue of transforming growth factor-beta 1 null mice.. Science.

[29] Aaltonen R, Heikkinen T, Hakala K, Laine K, Alanen A (2005). Transfer of proinflammatory cytokines across term placenta.. Obstet Gynecol.

[30] Reisenberger K, Egarter C, Vogl S, Sternberger B, Kiss H (1996). The transfer of interleukin-8 across the human placenta perfused in vitro.. Obstet Gynecol.

[31] Smith JT, Waddell BJ (2003). Leptin distribution and metabolism in the pregnant rat: transplacental leptin passage increases in late gestation but is reduced by excess glucocorticoids.. Endocrinology.

[32] Waysbort A, Giroux M, Mansat V, Teixeira M, Dumas JC (1993). Experimental study of transplacental passage of alpha interferon by two assay techniques.. Antimicrob Agents Chemother.

[33] Roth P, Dominguez MG, Stanley ER (1998). The effects of colony-stimulating factor-1 on the distribution of mononuclear phagocytes in the developing osteopetrotic mouse.. Blood.

